# The phenotypic spectrum of pathogenic *ATP1A1* variants expands: the novel p.P600R substitution causes demyelinating Charcot–Marie–Tooth disease

**DOI:** 10.1007/s00415-023-11581-w

**Published:** 2023-02-04

**Authors:** Feride Cinarli Yuksel, Paschalis Nicolaou, Kerri Spontarelli, Maike F. Dohrn, Adriana P. Rebelo, Pantelitsa Koutsou, Anthi Georghiou, Pablo Artigas, Stephan L. Züchner, Kleopas A. Kleopa, Kyproula Christodoulou

**Affiliations:** 1 Neurogenetics Department, The Cyprus Institute of Neurology and Genetics, 1683 Nicosia, Cyprus; 2 Department of Cell Physiology and Molecular Biophysics, Center for Membrane Protein Research, Texas Tech University Health Sciences Center, Lubbock, TX USA; 3 Dr. John T. Macdonald Foundation, Department of Human Genetics and John P. Hussman Institute for Human Genomics, University of Miami, Miller School of Medicine, Miami, FL USA; 4 Department of Neurology, RWTH Aachen University Hospital, Aachen, Germany; 5 Neuroscience Department and the Centre for Neuromuscular Disorders, The Cyprus Institute of Neurology and Genetics, 1683 Nicosia, Cyprus

**Keywords:** Charcot–Marie–Tooth, ATP1A1, Na^+^/K^+^ ATPase, Expression, Ouabain survival assay, Electrophysiology

## Abstract

**Background:**

Charcot–Marie–Tooth disease (CMT) is a genetically and clinically heterogeneous group of inherited neuropathies. Monoallelic pathogenic variants in *ATP1A1* were associated with axonal and intermediate CMT. *ATP1A1* encodes for the catalytic α1 subunit of the Na^+^/ K^+^ ATPase. Besides neuropathy, other associated phenotypes are spastic paraplegia, intellectual disability, and renal hypomagnesemia. We hereby report the first demyelinating CMT case due to a novel *ATP1A1* variant.

**Methods:**

Whole-exome sequencing on the patient’s genomic DNA and Sanger sequencing to validate and confirm the segregation of the identified p.P600R *ATP1A1* variation were performed. To evaluate functional effects, blood-derived mRNA and protein levels of *ATP1A1* and the auxiliary β1 subunit encoded by *ATP1B1* were investigated. The ouabain-survival assay was performed in transfected HEK cells to assess cell viability, and two-electrode voltage clamp studies were performed in Xenopus oocytes.

**Results:**

The variant was absent in the local and global control datasets, falls within a highly conserved protein position, and is in a missense-constrained region. The expression levels of ATP1A1 and ATP1B1 were significantly reduced in the patient compared to healthy controls. Electrophysiology indicated that *ATP1A1*^p.P600R^ injected Xenopus oocytes have reduced Na^+^/ K^+^ ATPase function. Moreover, HEK cells transfected with a construct encoding *ATP1A1*^p.P600R^ harbouring variants that confers ouabain insensitivity displayed a significant decrease in cell viability after ouabain treatment compared to the wild type, further supporting the pathogenicity of this variant.

**Conclusion:**

Our results further confirm the causative role of *ATP1A1* in peripheral neuropathy and broaden the mutational and phenotypic spectrum of *ATP1A1*-associated CMT.

**Supplementary Information:**

The online version contains supplementary material available at 10.1007/s00415-023-11581-w.

## Introduction

Charcot–Marie–Tooth (CMT) disease is a group of hereditary motor and sensory neuropathies (HMSN) characterized by high genetic and phenotypic heterogeneity [1]. CMT is the most common inherited neuropathy, with a reported global incidence of 1 in 2500 people [2]. CMT is classified into demyelinating (CMT1), axonal (CMT2) or intermediate (CMTI) types based on the neurophysiological criteria. To date, variants in over 120 genes have been linked to CMT pathology.

Pathogenic variants in *ATP1A1* (ATPase Na^+^/K^+^ Transporting Subunit Alpha 1) were previously associated with peripheral neuropathy. The *ATP1A1* gene encodes for the catalytic α1-subunit of the Na^+^/ K^+^ ATPase that actively transports the Na^+^ and K^+^ ions against their concentration gradients across the cell membrane using the energy of ATP hydrolysis. The pump activity is critical for maintaining resting membrane potential, cell excitability, uptake of nutrients, amino acids, and neurotransmitters, and for maintaining cell volume and intracellular pH. There are four α-subunit isoforms (α1–α4) of the Na^+^/K^+^ ATPase encoded by distinct genes that share sequence similarity of more than 75% [3]. ATP1A1 protein is expressed ubiquitously, including in the neurons and glial cells of the brain. Mammals express three auxiliary β subunits (β1–β3). *ATP1B1* encoded β1 subunit is expressed almost in all tissues and cells whereas β2 and β3 have restricted tissue expression patterns [4–8]. The β subunit is required to target the Na^+^/K^+^ ATPase to the plasma membrane. It also acts as an intracellular adhesion protein by interacting with other β subunits of neighbouring cells [9]. A functional pump is formed by combining one α-, one β-, and optionally one of seven regulatory FXYD subunits [10]. α1 and β1 are the most common heterodimer constituents considered to form the housekeeping Na^+^/ K^+^ ATPase isozymes [11].

Variants in *ATP1A1,* but no other α paralogs were associated with CMT disease to date. Missense variants were identified in *ATP1A1* (p.L48R, p.I592T, p.A597T, p.P600T, p.P600A, p.D601F, and p.D811A) in distinct pedigrees affected with autosomal dominant CMT2 [12]. Moreover, two missense variants at positions p.S207F and p.G877S were identified in two Chinese families, and p.G549R was identified in a Spanish proband with intermediate CMT [13, 14].

We identified a novel variant in *ATP1A1* that substitutes proline at position 600 for arginine (p.P600R) in a demyelinating CMT patient of Cypriot origin. The variant maps to the previously identified hotspot region of pathogenic axonal CMT variants in *ATP1A1* [12]. In this study, we investigated the patient and control blood expression levels of α1 and β1. Furthermore, electrophysiological properties of mutant ATP1A1 versus wild type ATP1A1 were investigated in Xenopus oocytes. Finally, a sodium pump-specific cell viability assay was performed in HEK cells to further characterize the deleterious effects of this novel CMT1-related *ATP1A1* variant.

## Materials and methods

### Patient

Clinical and neurophysiological data were collected from the patient through detailed medical evaluation. Peripheral blood was collected for molecular analysis from the patient and participating controls, including the patient’s healthy brother.

### Genetic analysis

#### Screening of the common CMT1 and CMT4 genes

DNA extraction from whole blood and then targeted gene analysis were performed. Briefly, the duplication of *PMP22* was evaluated by Multiple Ligation-Dependent Probe Amplification (MLPA) using the SALSA MLPA kit (MRC-Holland, Amsterdam, Netherlands) according to the manufacturer’s instructions. The results were analysed on the Coffalyser software (MRC-Holland). For Sanger sequencing, primers were designed using the Primer3 software to amplify the coding regions of *Cx32, PMP22, MPZ, EGR2, NEFL* and *GDAP1* by standard PCR. Purified PCR products were sequenced with the BigDye Terminator V.1.1 Cycle Sequencing Kit (ABI, California, USA) and run on a 16-capillary ABI 3130XL sequencer. Sequencing analysis was performed using the SeqScape^®^ Software (ABI, California, USA) by aligning the sample sequence against the reference sequence obtained from the Ensembl genome browser database, human genome assembly: GRCh38p.13 (https://www.ensembl.org/index.html).

#### Whole-exome sequencing

Whole-exome sequencing (WES) was performed on genomic DNA obtained from the patient’s peripheral blood as previously described [15]. The DNA library was prepared using the Illumina’s TruSeq DNA Exome Kit. Sequencing was performed on the NextSeq-500 platform using the NextSeq 550 High Output kit (150 cycles) according to the manufacturer protocol (Illumina, California, USA). Raw sequence reads were aligned to the human genome reference sequence hg19 using FastQC 0.11.7 and BWA (Aligner) 0.7.15. Imported variants were annotated, filtered, and classified using VariantStudio (Illumina, California, USA). The variants were further filtered using the dbSNP (https://www.ncbi.nlm.nih.gov/snp/) and the Genome Aggregation Database (https://gnomad.broadinstitute.org/). Sanger sequencing was performed to confirm the presence of the NGS-derived variant in the patient. Familial segregation analysis by Sanger sequencing was limited to the patient’s non-affected brother due to the unavailability of parents or other family members.

#### In silico predictions

A total of five publicly available in silico tools were utilised for predicting variant pathogenicity and conservation of the substituted amino acid, including the Mutation taster (http://www.mutationtaster.org/), PROVEAN (http://provean.jcvi.org/index.php), PolyPhen-2 (http://genetics.bwh.harvard.edu/pph2/), PANTHER (http://www.pantherdb.org/tools/), CADD (https://cadd.gs.washington.edu/snv).

#### qPCR analysis of gene expression in blood

The total RNA was extracted from the peripheral blood obtained from the patient and healthy controls using the Nucleospin RNA Blood kit (Macherey–Nagel, Duren, Germany). Reverse transcription was performed using the Protoscript cDNA Synthesis Kit (NEB, Massachusetts, USA) according to the manufacturer’s instructions. The starting quantity of 2.5 ng cDNA was determined by the standard curve assay performed on the Quant Studio 7 Flex System (ABI, California, USA). Primers specific to *ATP1A1* and *ATP1B1* were used, and normalisation was achieved with housekeeping genes GAPDH and β-Actin (Supplementary Table 1). Primers were designed using primer3 to span exon–exon junctions to prevent amplification from the genomic DNA. Primers’ specificities were confirmed using the Primer-Blast software tool (https://www.ncbi.nlm.nih.gov/tools/primer-blast/). The samples were analysed in triplicates in each run, and three independent experiments were performed.

#### Sequencing of cDNA derived from blood RNA

We investigated whether the observed reduction of *ATP1A1* gene expression was due to the absence of the mutant *ATP1A1* transcript in the patient blood. The total RNA extracted from patient blood was converted to cDNA. 200 ng cDNA template was amplified by PCR using a set of *ATP1A1*-specific primers (Forward: ATP1A1_Exon_6_7F; Reverse: ATP1A1_Exon_14R). Agarose gel electrophoresis was performed to verify the correct amplicon size of the PCR product and the product was sequenced with the primers used for PCR amplification. Sequencing analysis was performed on SeqScape software as described above.

### Cell culture

#### Lymphoblastoid cell line (LCLs) cultures

LCLs were chosen for functional analysis since *ATP1A1* expression is not detectable in fibroblasts. Patient and control lymphocytes were collected from peripheral blood using Ficoll–Paque Plus upon obtaining written informed consent (Sigma-Aldrich, USA). Selected lymphocytes were infected with the Epstein–Barr virus (EBV) and were cultured in RPMI 1640 medium (Biosera, Nuaille, France), supplemented with 10% FBS (ThermoFisher, Massachusetts, United States), and 50 U/ml of Penicillin/Streptomycin (Biosera) as previously described [16].

#### Protein extraction

Total protein was isolated from lymphoblastoid cells by cell lysis (50 mM Tris–Cl pH 7.5, 150 mM KCl, 2 mM EDTA, 0.5% Triton x-100 and 1 × protease inhibitor cocktail) followed by one-hour incubation on ice. Cell lysates were sonicated and centrifuged at 4 °C. Protein concentration was determined using the Coomassie Plus (Bradford) protein assay (ThermoFisher Scientific, UK).

### Immunoblotting

Ten μg of protein from each lymphoblastoid cell lysate was treated with SDS in the presence of 10% β-mercaptoethanol for denaturation and loaded onto an 8–10% SDS-PAGE gel for separation on a Mini-Protean system (Bio-Rad) and ran at 90 mV for ~ 20 min, then 120 mV until desired migration was achieved. Proteins were transferred onto the Immobilon P-polyvinylidene difluoride (PVDF) membrane by electroblot at a constant 100 mV for 1 h. After the transfer was complete, the PVDF membrane was blocked in PBS containing 0.1% v/v Tween-20 and 5% BSA for one hour. The membranes were incubated overnight at 4 °C with the respective primary antibody. This was followed by three 5–15 min washes in PBS with 0.1% v/v Tween-20. Then, the membranes were incubated with the appropriate horseradish-peroxidase conjugated secondary antibody for 1–2 h at room temperature. Membranes were washed three times in PBS with 0.1% Tween-20 and then incubated with Clarity Western ECL Substrate (Bio-Rad, USA). Visualization of protein bands was processed in the Vilber Fusion Solo X imaging system. Protein expression levels were quantified using the ImageJ software.

### Antibodies

Primary antibodies of mouse anti-Na^+^/K^+^-ATPase A1/C464.6: sc-21712, Santa-Cruz (1:200), mouse anti-Na^+^/K^+^-ATPase β1 (C464.8): sc-21713, Santa-Cruz (1:200), mouse anti-GAPDH/sc-32233, Santa-Cruz (1:10,000) and mouse Anti-β-Actin, Clone AC-74 (A2228), Sigma (1:20,000) were used.

Secondary antibodies of donkey anti-mouse IgG (H + L) SA1-100/Invitrogen (1:10,000) and donkey anti-rabbit IgG (H + L) 31,458/Invitrogen (1:10,000) were used.

### Site-directed mutagenesis and heterologous expression in oocytes

P600R was introduced by PCR mutagenesis into the full-length cDNA encoding the ouabain-resistant human α1-isoform (with the mutations Q118R and N129D that mimic the naturally ouabain-resistant rodent α1 isoforms) [17]. For simplicity we refer to the “RD” mutant template as wild type. The resulting plasmid was sequenced, linearized via restriction enzyme (NdeI; New England Biolabs), and transcribed (SP6 Transcription Kit; Thermo Fisher) for injection into *Xenopus* laevis oocytes. Ovarian follicles (Xenoocyte) were enzymatically digested as previously described [18]. The cRNA of either P600R α1 or wild-type α1 was mixed with cRNA for the β1-isoform at an equimolar ratio and injected into oocytes (75 ng α, 25 ng β). Oocytes were stored at 16 °C in SOS solution (100 mM NaCl, 1 mM MgCl2, 2 mM KCl, 1.8 mM CaCl2, 5 mM HEPES, 2.5 mM pyruvic acid (Sigma-Aldrich), 1 × antibiotic–antimycotic (Gibco), and 5% horse serum (Gibco), titrated to pH 7.5 with NaOH) for 3–7 days.

### Electrophysiology

To increase the rate-limiting intracellular Na^+^ concentration oocytes were incubated in Na^+^-loading solution (in mM, 90 NaOH, 20 TEA-OH, 40 HEPES, and 0.2 EGTA) with 10 µM ouabain one hour before the two-electrode voltage clamp (TEVC) experiments. Such ouabain concentration inhibited endogenous pumps for the duration of the experiments leaving exogenous pumps unaffected [19].

TEVC was performed with an OC-725C amplifier (Warner Instruments) controlled by pClamp software and a Digidata 1440A (Molecular Devices). Current signals were acquired at 10 kHz and also continuously recorded at 1 kHz via a Minidigi 1B (Molecular Devices). The current and voltage glass microelectrodes were filled with 3 M KCl and had resistances of 0.2–1 MΩ. Oocytes were clamped at a holding potential of − 50 mV from which square voltage steps were applied as indicated.

Experiments were carried out at room temperature 19–21 °C in an extracellular solution containing 122 mM methanesulfonic acid (MS), 5 mM Ba(OH)_2_, 1 mM Mg(OH)_2_, 0.5 Ca(OH)_2_, 10 mM HEPES, 125 mM NaOH (titrated to pH 7.6 with MS, osmolality of 250–260 mOsm/kg). K^+^ was added to the external solution from a 450 mM K-MS stock. Ouabain (Sigma-Aldrich) was directly dissolved at 10 mM in the external solutions to reversibly inhibit the ouabain-resistant exogenously expressed pumps.

### Data analysis

Electrophysiological data was analyzed using Clampfit (part of pClamp software; Molecular Devices) and Origin (OriginLab). Na^+^/K^+^ pump-mediated transient currents were calculated subtracting the current induced by voltage pulses in the presence of ouabain from the current in the absence of ouabain. Only the current when the currents return to the baseline was analysed by subtracting the baseline current and integrating the transient current to obtain the charge moved during the voltage pulse. The charge–voltage relationship was fitted with the Boltzmann equation:

$${\text{Q}} = {\text{Q}}_{{{\text{hyp}}}} - {\text{Q}}_{{{\text{tot}}}} /\left( {\left[ {{1} + {\text{exp }}\left( {{\text{z}}_{{\text{q}}} {\text{e}}\left( {{\text{V}} - {\text{V}}_{{{1}/{2}}} } \right)} \right)/{\text{kT}}} \right]} \right),$$

where the charge moved by hyperpolarizing pulses is Q_hyp_, the total charge moved is Q_tot_, the voltage at the centre of the distribution is V_1/2_, the apparent valence of a charge crossing the whole electric field is z_q_, the elementary charge is e, the Boltzmann constant is k, the absolute temperature is T (°K). kT/ez_q_ is commonly referred to the slope factor.

The K^+^ concentration dependence of the current was fitted by a Hill function:

$${\text{I}} = {\text{I}}_{0} + {\text{ I}}_{{{\text{Max}}}} \left( {\left[ {{\text{K}}^{ + } } \right]^{{\text{n}}} /\left( {{\text{K}}^{{\text{n}}}_{{0.{5}}} + \, \left[ {{\text{K}}^{ + } } \right]^{{\text{n}}} } \right)} \right),$$

where I_0_ is the current at 0 K^+^, I_Max_ is the current at infinite [K^+^], K_0.5_ is the half maximal activating [K^+^], n is the Hill coefficient.

### Ouabain survival assay

Cells were transfected with plasmids encoding full-length human *ATP1A1* (WT), and an *ATP1A1* construct that has been mutated to be ouabain-insensitive (WT-oua). Cells transfected with plasmid constructs to express wild-type (WT-oua) and selected mutants (p.P600R-oua, p.G509D-oua, p.G718S-oua) were treated with 0.5 μM ouabain over 72 h. The p.G509D and p.G718S mutants were included as positive controls, involved in a complex neurodevelopmental syndrome, and were included in this analysis due to their proximity to the p.P600 variant. Cell survival was measured using the CellTiter-Glo luciferase assay.

### Statistical analysis

WB: Quantitative data from at least three independent experiments were analysed with one-way ANOVA. *P* value < 0.05 was considered as statistically significant. Results are expressed as mean% ± SE (Standard error) from the three independent experiments. The mean quantitative data of the controls was normalised to 100%.

OSA: Experiments were conducted in replicates of eight, and survival of a specific mutant was normalized to untreated cells transfected with the same plasmid. Normalized cell survival under ouabain treatment was compared by ANOVA, using the Tukey’s post-test for alpha-error correction.

## Results

### Patient phenotype

The proband is a 50-year-old female who first presented with progressive leg and hand weakness at the age of 5 years. Nerve conduction studies performed at the age of 12 years showed prolonged ulnar and median motor nerve distal latencies (median: 5.2 ms; ulnar: 5.6 ms) and slowing of motor nerve conduction velocities (MNCVs) between 22.2 m/s (ulnar) and 27.4 m/s (median), with low compound muscle action potential (CMAP) amplitudes (median: 1.9 mV; ulnar: 3.0 mV) showing temporal dispersion. Motor responses in the lower limbs and sensory responses in both upper and lower limbs (median ulnar, sural) were all unobtainable. Median and ulnar F-wave responses were also abolished. Selective needle electromyography showed severely reduced recruitment of high amplitude, long duration, polyphasic motor unit potentials and spontaneous activity present in the tibial anterior, peroneus longus and medial gastrocnemius muscles, consistent with severe chronic-active neurogenic process.

At age 14, she had a tenotomy of the Achilles tendon, while she slowly developed more proximal muscle weakness and started having gait dysfunction after adolescence. In parallel, she developed weakness of the hand muscles, atrophy of all distal muscles and decreasing function in both arms and legs.

At the age of 43, the patient reported having difficulties climbing stairs in recent years and stated that she felt much weaker overall. At last examination, she needed help with all activities of daily living. Her family history is negative for any neuromuscular disease. She had a healthy older brother, and both of her deceased parents were reported to be neurologically unaffected.

The patient had severe bilateral sensorineural hearing loss, weakness and atrophy of all distal limb muscles (1–2/5 MRC scale distal to the elbows and knees), mild-moderate weakness of proximal muscles (4 + upper limbs, 4 to 4− lower limbs), areflexia, and severe sensory loss in a stocking-glove distribution. She walked with an unsteady steppage gait and impaired balance. The CMT neuropathy score (CMTNSv2) was 34.

Electrodiagnostic studies at that time revealed unobtainable motor nerve responses from upper and lower limbs with the exception of radial motor response showing prolonged distal latency (6.1 ms), reduced CMAP amplitude (0.1 mV) and severe slowing of the MNCV (8.0 m/s). Severe chronic denervation was detected by needle EMG in the triceps brachii muscle. Further neurophysiological evaluation with brainstem auditory evoked potentials (BAEP) confirmed severe peripheral hearing loss, while visual evoked responses were normal.

In the absence of family history and based on her neurological and electrodiagnostic findings, the clinical diagnosis was established as demyelinating CMT type 1 or 4.

### Identification of the novel ATP1A1 c.1799 C > G missense variant in the CMT1 patient

**Fig. 1:**
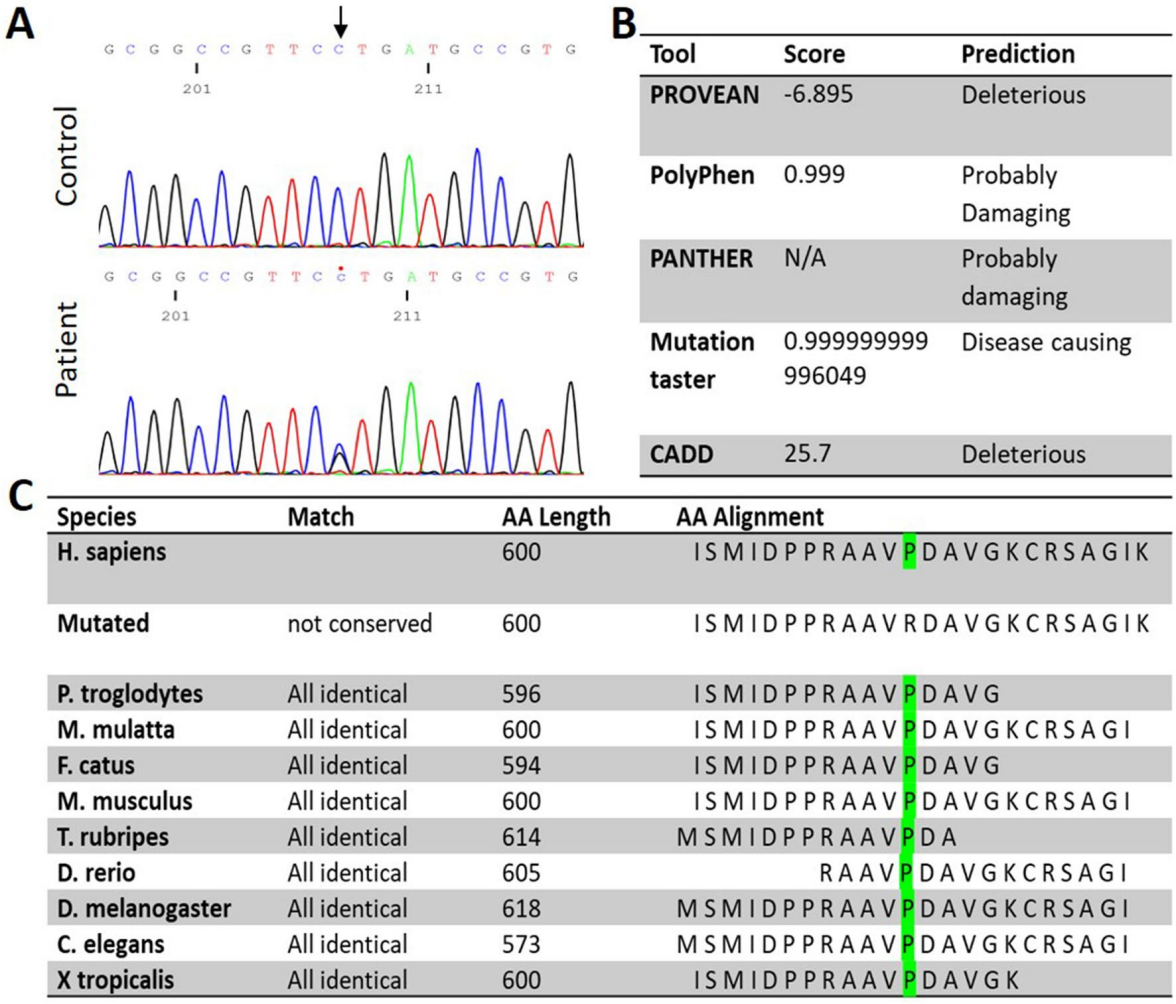
Genetic findings of the Cypriot proband. **A** Electropherogram of the control *ATP1A1* sequence (upper) versus patient *ATP1A1*: c.1799C > G (p.P600R) sequence (lower). **B** In silico analysis of the *ATP1A1*: c.1799C > G (p.P600R). **C** Conversation analysis of amino acid sequences of ATP1A1 (NM_000701.8) across species compared to p.P600R

MLPA analysis of *PMP22* duplication and Sanger sequencing of the coding regions of the most common CMT genes; *Cx32, PMP22, MPZ, EGR2, NEFL,* and *GDAP1* were negative in the patient. WES has identified a heterozygous missense variant, c.1799C > G (NM_000701.8), in exon 13 of *ATP1A1*. Sanger sequencing confirmed the variant in the patient, whereas her healthy brother was found to be negative (Fig. 1A). The variant was absent in the large dataset of GnomAD and the in-house dataset (100 WES) of the Cypriot population. The amino acid sequence at the position of the variant was highly conserved, affecting a highly constrained region, and predicted to be damaging according to the in silico prediction tools (Fig. 1B, C). A CADD score of 25.7 further suggested the deleteriousness of this variant. The 67 bp region including the p.600P in *ATP1A1* has a constraint coding region (CCR) score of ≥ 99th percentile, indicating that the region is under purifying selection and is highly constrained for genomic variation (https://s3.us-east-2.amazonaws.com/ccrs/ccr.html) [20]. The inheritance pattern of the variant could not be investigated due to the absence of parents who were reported to be neurologically unaffected. In addition, the negative family history for any neuromuscular disorder suggests that the patient could have acquired the variant de novo.

### RNA and protein expression analysis in the CMT1 patient and controls

#### ATP1A1 expression

**Figure 2:**
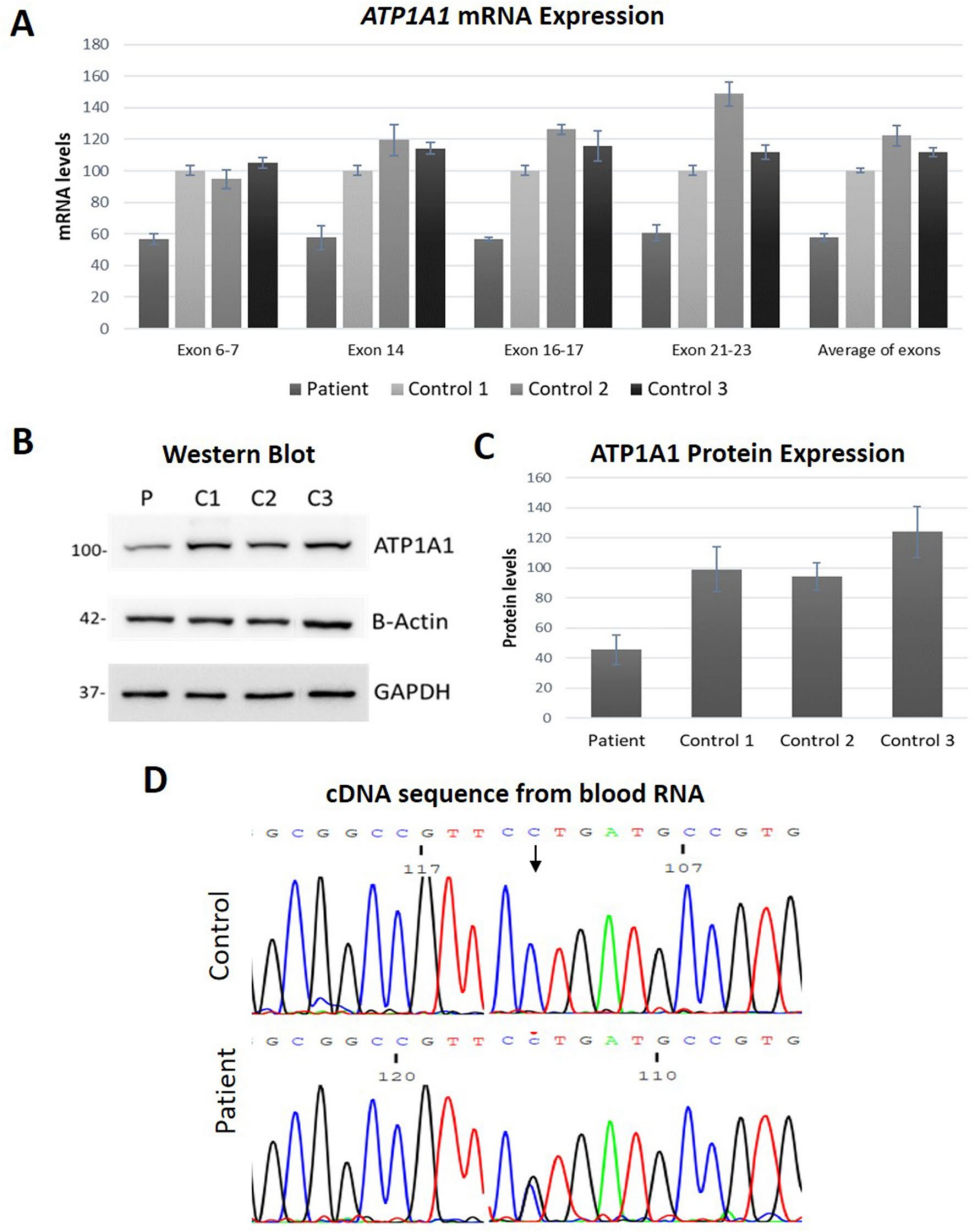
mRNA and protein expression analysis of the wild type and mutant *ATP1A1*. **A** mRNA expression of *ATP1A1* exons in the patient peripheral blood cells compared to healthy controls (Patient: 58.04 ± 2.16%, Control 1: 100.25 ± 2.32%, Control 2: 122.32 ± 6.54%, Control 3: 111.76 ± 2.77%. *P* = 0.00002). Three independent qPCRs were performed and samples were run as triplicates in each experiment. **B** Western blot analysis results of ATP1A1 expression normalized against β-ACTIN and GAPDH. **C** Quantification of protein expression of ATP1A1 in the patient versus healthy control LCLs (Patient: 45.49 ± 10.02%, Control 1: 99.01 ± 14.84%, Control 2: 94.15 ± 9.10%, Control 3: 123.94 ± 17.00%. *P* = 0.002). WB was replicated eight times for quantification. Statistical analysis was performed by one-way ANOVA. *P* < 0.05 considered statistically significant. **D** The expression of mutant *ATP1A1* (c.1799 C > G, P600R) transcript in patient blood confirmed by Sanger sequencing

Relative mRNA and protein expression was investigated by performing qPCR analysis and Western blotting. The results of the qPCR analysis indicated a reduction in the expression of *ATP1A1* mRNA to approximately 50% in patient blood compared to healthy controls (Fig. 2A). Western blot analysis performed in patient-derived lymphoblasts further confirmed the significant reduction of ATP1A1 protein expression in the CMT1 patient compared to healthy controls (Fig. 2B, C).

#### ATP1A1 P600R transcript detection

RNA from patient blood was converted into cDNA and used as a template for *ATP1A1* sequencing of the region containing the c.1799C > G change (Fig. 2D). The mutant transcript was expressed in equal level with the wild type transcript in the patient, indicating that the observed reduction of *ATP1A1* transcript levels in lymphoblastoid cells is not an mRNA synthesis defect due to the c.1799C > G variant.

#### ATP1B1 expression

**Figure 3:**
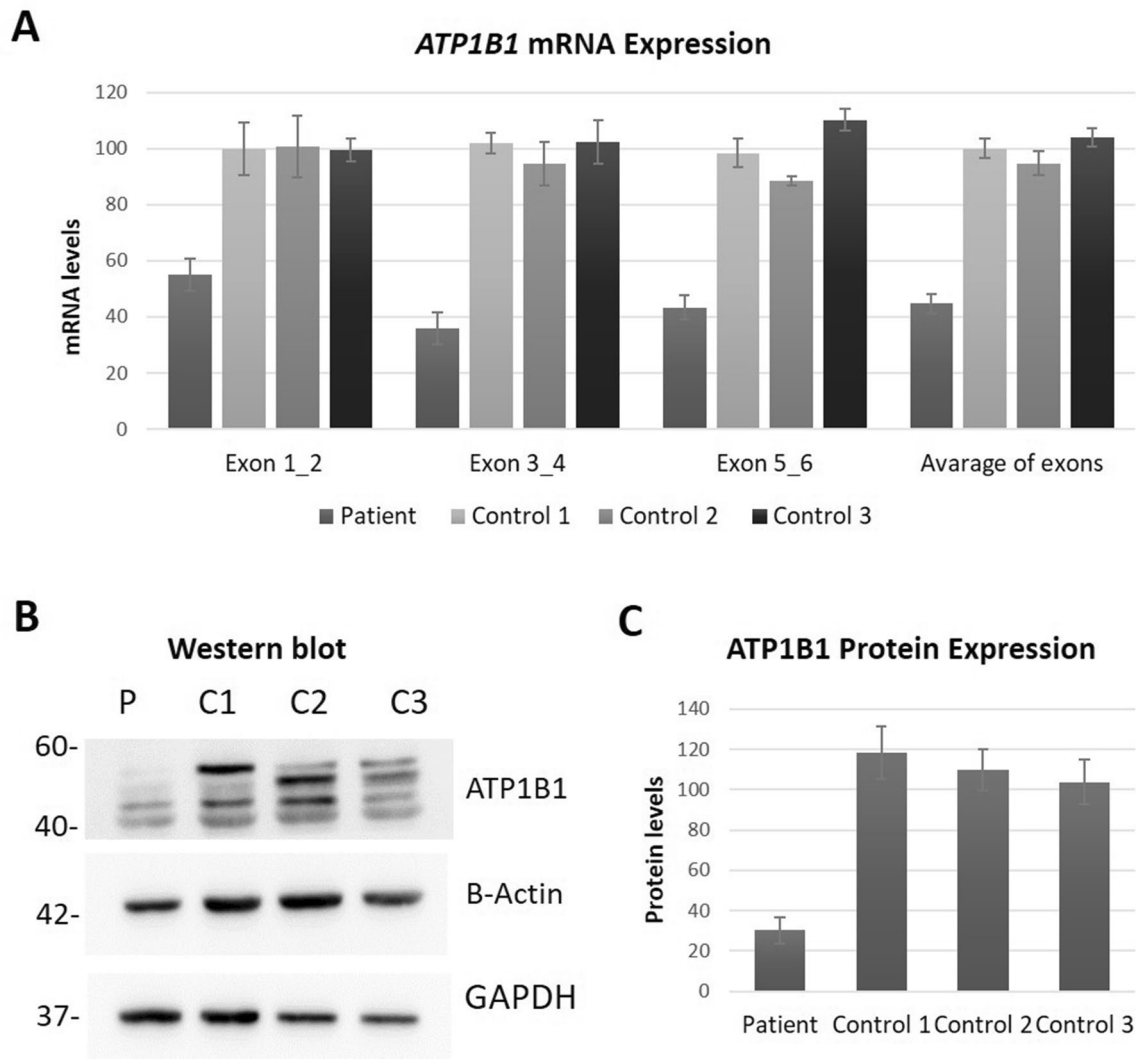
mRNA and protein expression analysis of *ATP1B1*. **A** mRNA expression analysis of *ATP1B1* cDNA in the patient peripheral blood cells compared to healthy controls (Patient: 44.71 ± 3.63%, Control 1: 100.04 ± 3.40%, Control 2: 94.63 ± 4.31%, Control 3: 103.99 ± 3.21%. *P* = 0.00001). Four independent qPCRs were performed and samples were analysed as triplicates in each run. **B** Western blot analysis results of ATP1B1 expression normalized against β-ACTIN and GAPDH. **C)** ATP1B1 protein expression quantification in patient versus healthy control LCLs (Patient: 30.22 ± 6.43%, Control 1: 118.20 ± 13.21%, Control 2: 110.05 ± 10.23%, Control 3: 103.64 ± 11.04%. *P* = 0.0002). WB was replicated four times for quantification. Statistical analysis was performed by one-way ANOVA. *P* < 0.05 considered statistically significant

We investigated the *ATP1B1* expression levels in the patient blood due to its ubiquitous expression and the most prevalent associate of the α1 subunit amongst other β subunits. The β1 subunit contains N-glycosylated forms in the range of ~ 60–40 kDa consisting of mature, hybrid and immature types [21] (Fig. 3B). Investigation of ATP1B1 mRNA and protein levels were performed by qPCR and Western blot analysis, respectively. *ATP1B1* mRNA was reduced to ~ 45% in patient blood in comparison to healthy controls (Fig. 3C). The protein levels of β1 subunit in LCLs were further reduced to ~ 30% in the patient compared to healthy controls.

### Electrophysiology

**Fig. 4:**
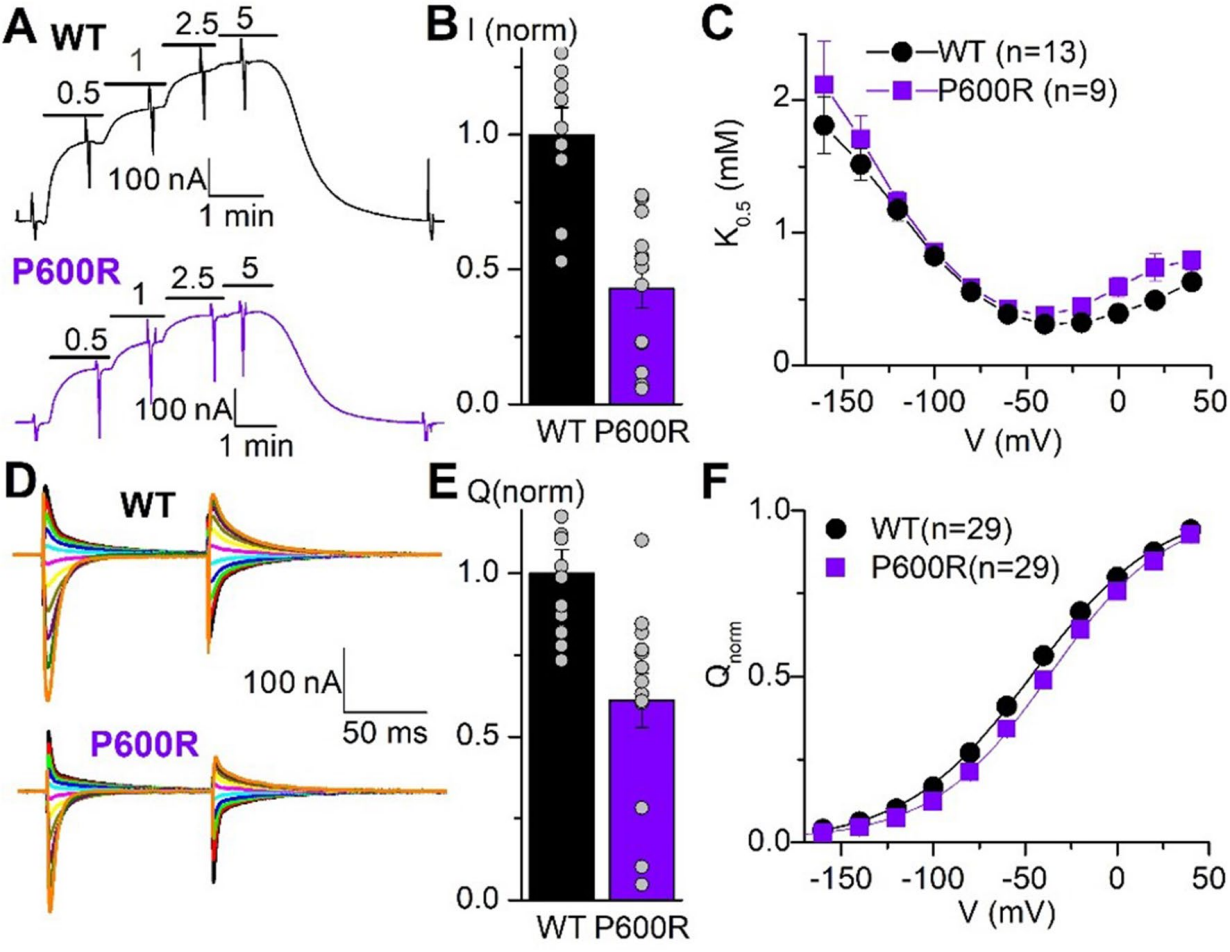
Electrophysiological findings. **A** Representative traces from an oocyte expressing WT (that is the RD ouabain resistant version) and P600R pumps. Oocytes were held at − 50 mV and exposed to the indicated [K^+^] (in mM) to activate pump current. Vertical deflections indicate application of 100 ms-long pulses to measure voltage-dependent parameters. **B** K_0.5_ for K^+^ activation of pump current obtained from Hill fits (“Methods”) to the [K^+^] dependencies at each voltage, as a function of the applied voltage. **C** Current, normalized to the mean from oocytes expressing WT pumps on the same day under the same conditions (results from 3 batches of oocytes, 11 WT and 13 P600R oocytes total. **D** Ouabain-sensitive transient currents elicited by pulses from − 50 mV to voltages ranging from − 140 mV to + 40 mV (in 20 mV increments). **E** Charge (integral of current transients) as a function of voltage normalized to the total charge moved in the whole voltage range (*Q*_tot_ = 21.6 ± 10.6 for WT and 15.3 ± 11.1 for P600R). Line plots are Boltzmann distributions fitted to the data (mean *V*_0.5_ = − 47.5 ± 4.3 mV for WT and − 38.1 ± 4.3 mV for P600R, *n* = 29 each). **F** Mean total charge (measured from Boltzmann distributions in individual experiments) normalized to the mean from oocytes expressing WT pumps on the same day

TEVC was performed in WT (ouabain resistant) versus mutant P600R *ATP1A1* injected Xenopus oocytes to determine the electrophysiological characteristics of the pump function (Fig. 4). The concentration dependence of outward current activated by K^+^ (Fig. 4A) illustrates the reduced maximal pump current (Fig. 4B), without alteration of the K_0.5_ for K^+^. The maximal pump current is proportional to both the number of pumps and their turnover rate. A better estimation of the number of functional pumps in the plasma membrane can be obtained by measuring the total charge moved in transient currents elicited by voltage pulses in the absence of K^+^ (Fig. 4D). As these transient currents report on the conformational change of the pump, without cycling, the total charge moved across the whole voltage range is only proportional to the number of pumps (independent of the turnover rate). Upon integration of the transient currents the mean total charge from Boltzmann fits to individual experiments was *Q*_tot_ = 21.6 ± 10.6 nC for WT and 15.3 ± 11.1 for P600R (*n* = 29, SD for both). The *Q*_tot_ from P600R-injected oocytes normalized to WT-injected oocytes injected and measured on the same days, was reduced by about 50% (Fig. 4E). Effects of a mutation on the apparent affinity for external Na^+^ can be estimated from the centre of the Boltzmann distribution in the *Q*–*V* curves [18, 19] (Fig. 4F). The minor ~ 10 mV rightward shift induced by P600R indicates a 50% increase in affinity for external Na^+^, that is not expected to be of pathological significance. Thus, the results in *Xenopus* oocytes suggest that the pathological effects of the P600R mutation are not due to changes in affinities for Na^+^ or K^+^.

### Functional effect of P600R variant on cell viability

**Fig. 5:**
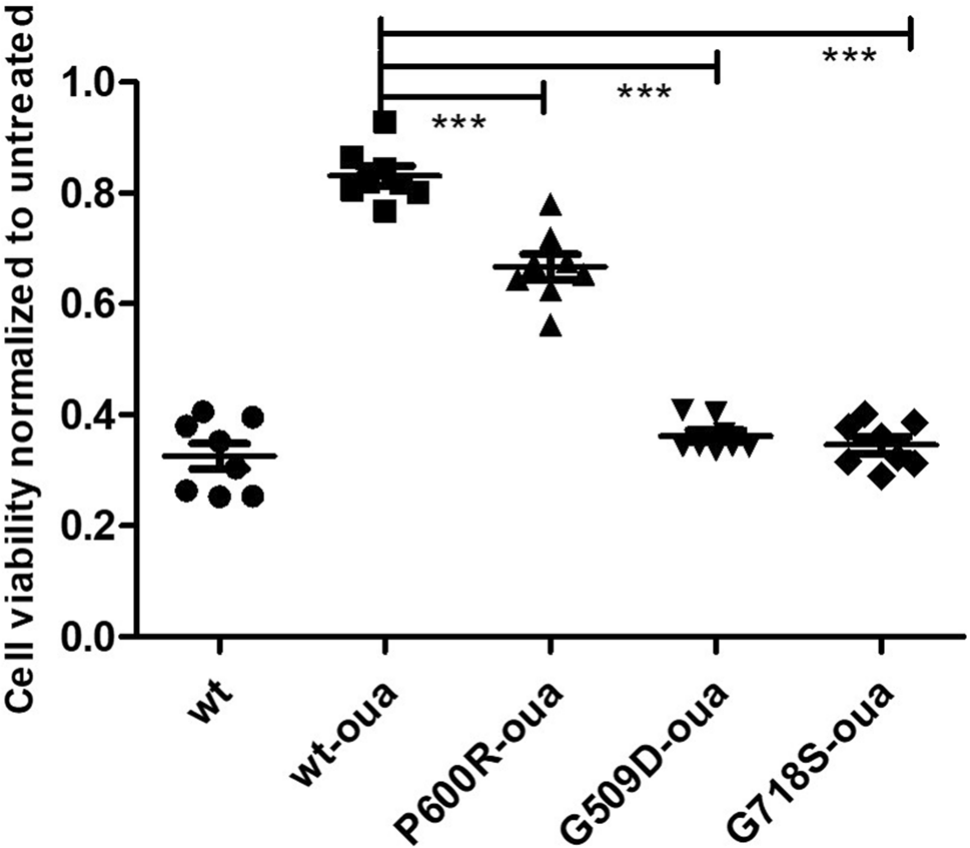
Ouabain survival (luciferase) assay. Viability of ouabain insensitive HEK cells transfected with wild type ATP1A1, p.P600R and, positive controls p.G509D and p.G718S normalized to untreated cells transfected with the same plasmid

The functional effect of the c.1799 C > G (p.P600R) variant was further evaluated with an ouabain survival assay using HEK cells transfected with ouabain-insensitive ATP1A1 constructs as described [12]. Cell death was observed in the cells transfected with WT *ATP1A1*, whereas cells transfected with the ouabain-resistant WT (WT-oua) survived. Cells transfected with selected mutants demonstrated a significant decrease in viability in all groups. Cells transfected with p.G509D-oua and p.G718S-oua, two positive controls described in association with a severe neurodevelopmental syndrome, showed a higher cell death proportion than the p.P600R-oua (Fig. 5).

## Discussion

We present a Cypriot patient with early-onset, progressive demyelinating CMT accompanied by severe sensorineural hearing loss. WES identified a novel c.1799C > G (p.P600R) missense change in *ATP1A1* in this patient. This variant was absent in the general and local population datasets. The ancestral amino acid is highly conserved and its region is highly constrained for missense variants. In silico pathogenicity prediction tools suggested a damaging consequence for the p.P600R variant. The absence of this variant in the healthy brother and the negative family history for any neuromuscular disease suggests that the patient acquired the c.1799C > G variant de novo.

**Fig. 6:**
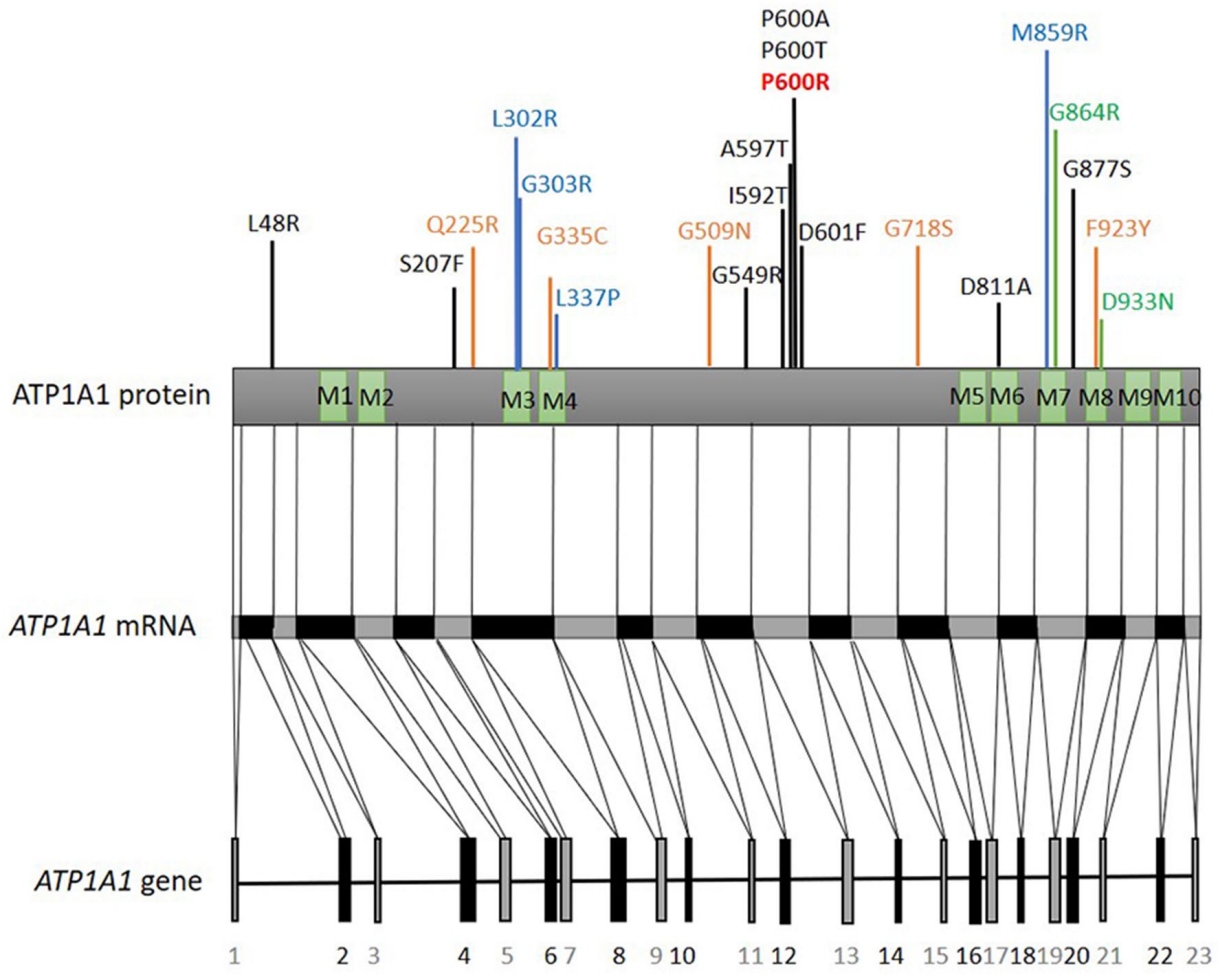
ATP1A1 protein and respective locations of the pathogenic variants reported up to date. Variants written in black represent CMT related variants identified in other studies, red represents the ATP1A1 variant identified in this study, blue represent hypomagnesaemia and intellectual disability or spastic paraplegia, green represent developmental delay, orange represent complex neurodevelopmental syndrome

The clinical findings in this case expand the spectrum of previously reported phenotypes associated with *ATP1A1* variants. Although intermediate slowing of NCV has been described [12] no MNCVs below 30 m/s were reported. In our patient, upper limb MNCVs were clearly in the demyelinating range, with only moderately reduced CMAP amplitudes in childhood. This may be explained by the fact that in addition to axons, ATP1A1 has been shown to be prominently expressed at the non-compact myelin areas of myelinating Schwann cells including the paranodal loops and Schmidt-Lantermann incisures [12]. Moreover, ATP1A1 and ATP1A3 showed mutually exclusive expression patterns throughout the spinal cord and ATP1A1 was strongly expressed in the myelin sheath of lumbar roots. Thus, it is plausible that dysfunction of this subunit can cause disturbed homeostasis starting in Schwann cells, with concurrent or secondary axonal degeneration, consistent with the phenotype in this case. Similar mechanisms have been described in other CMT genes associated with phenotypes spanning the axonal to intermediate and demyelinating range in terms of electrophysiology, as in CMT1X caused by variants in *GJB1*/Connexin32 gene also expressed at non-compact myelin [22]. Another novel finding in this case was the sensorineural hearing loss. Although the onset is unclear from the patient’s history because of slow progression over the years, it was clearly documented at the age of 43. Hearing loss of variable severity may occur in patients with both axonal and demyelinating inherited neuropathies [23] and it is possible that it might have been underrecognized in previous reports of patients affected by *ATP1A1* variants, if not reported by the patients and no BAEPs have been performed. De novo* ATP1A1* variants in CMT are rare and have not been genetically confirmed (current study and ref 12). Previously, de novo* ATP1A1* variants were reported in hypomagnesemia, spastic paraplegia, sleep disorder, intellectual disability and epileptic encephalopathy and, complex neurodevelopmental syndrome [24–27], whereas, inherited *ATP1A1* variants were associated with axonal or intermediate CMT [12–14] (Table 1). Six out of ten (60%) CMT2-ATP1A1 variants map to the large cytoplasmic loop between transmembrane domains M4 and M5, which contains the ATP binding (N) and the phosphorylation (P) site (Fig. 6). Two of these variants alter proline at position 600 to alanine or threonine. Here, we report a demyelinating CMT case due to a novel hotspot *ATP1A1* variant, p.P600R.

**Table 1:** Genetic and clinical features of CMT related *ATP1A1* variants

Case	Origin	AOO	Clinical diagnosis	Genetic diagnosis	Inheritance	Molecular findings	References
1	Czech	12–50 years	CMT2	c.143T > G (p.L48R)	AD	Significant loss of cell viability in ouabain survival assay	[12]
2	Italy	13–50 years	CMT2	c.1798C > G (p.P600A)	AD	Reduced Na^+^ dependent currents	[12]
3	USA	N/A	CMT2 with migraine	c.1775T > C (p.I592T)	AD	Significant loss of cell viability in ouabain survival assay	[12]
4	USA	12–31 years	CMT2 with headaches, GI and pulmonary issues	c.1798C > A (p.P600T)	AD	Significant loss of cell viability in ouabain survival assay	[12]
5	Australia	N/A	CMT2	c.1789G > A (p.A597T)	AD	N/A	[12]
6	Australia	8–36 years	CMT2	c.1801_1802delinsTT (p.N601F)	AD	N/A	[12]
7	Korea	18 years	CMT2 with gait ataxia	c.2432A > C (p.N811A)	N/A	Reduced Na^+^ dependent currents Significant loss of cell viability in ouabain survival assay	[12]
8	Chinese	18–40 years	CMTI	c.620C > T (p.S207F)	AD	Reduced ATP1A1 protein expression	[13]
9	Chinese	23 years	CMTI	c.2629G > A (p.G877S)	Mosaicism detected in the mother	Reduced ATP1A1 protein expression	[13]
10	Spanish	N/A	CMTI	c.1645G > A (p.G549R)	AD	N/A	[14]
11*	Cypriot	5 years	CMT1 with severe sensorineural hearing loss	c.1799C > G (p.P600R)	N/A	Reduced mRNA and protein expression. Significant loss of cell viability in ouabain survival assay and reduced ATP1A1^P600R^ expression in Xenopus oocytes	Current study

*AD* autosomal dominant, *AOO* age of onset, *CMT1* Charcot–Marie–Tooth type 1 demyelinating, *CMT2* Charcot–Marie–Tooth type 2 axonal, *CMTI* Charcot–Marie–Tooth intermediate type, *N/A* data not available.

Analysis of *ATP1A1* expression revealed a ~ 50% downregulation of its mRNA and protein levels in the CMT patient’s blood compared to healthy controls. To investigate the mechanism of this downregulation, we sequenced the patient derived cDNA spanning the c.1799C > G variant region. Results revealed that the mutant allele was able to produce the transcript in a similar amount to the wild type allele without frameshift or exon-skipping, confirming that the observed reduction of *ATP1A1* expression is not due to reduced biosynthesis or a splicing defect of the mutant transcript. The *β* subunit of the Na^+^/K^+^ ATPase is important for stabilizing the *α* subunit and its export from the ER to the plasma membrane, hence preventing its degradation [21, 28, 29]. The *β* subunit is also able to modify ion transport properties of the catalytic *α* subunit thus, modulating the activity of its *α* counterpart [30–32]. We investigated whether the expression level of *ATP1B1*, the ubiquitously expressed form of *β* would have been affected due to reduced *ATP1A1* levels in our CMT patient. Indeed, we identified a significant downregulation of *ATP1B1* mRNA and protein levels in our CMT patient compared to controls, similar to *ATP1A1* expression levels. This may suggest a dominant negative effect of the p.P600R variant. Alternatively, another mechanism might be playing a role for the regulation of the Na^+^/K^+^ ATPase expression by affecting the transcript levels of its subunits.

Electrophysiology indicated reduced pump function of the p.P600R mutant compared to the ouabain-sensitive WT pump. The mutant had minor effects on pump kinetics in the Xenopus oocytes, which are unlikely to cause the observed loss-of-function, which is probably caused by the reduced expression of the mutant pump seen both in the patient and in the oocyte expression system. Furthermore, ouabain insensitive-p.P600R transfected HEK cells demonstrated a significant decrease in viability compared to wild type ouabain insensitive-ATP1A1 transfected cells, in support of the pathogenicity of this variant. A more substantial cell death was observed in the positive control cells transfected with the p.G509D and p.G718S variants associated with the early-onset complex neurodevelopmental syndrome [27]. The phenotype severity has been reflected in the cell survival ratio as the “milder” p.P600R CMT1 variant caused a relatively less detrimental effect on cell viability compared to clinically more severe positive controls. Pathogenicity of the p.P600 ATP1A1 variants was also shown in axonal/intermediate CMT patients. The p.P600T variant caused a significant decrease in cell viability and the p.P600A variant significantly reduced the Na^+^-pump currents in Xenopus oocytes, indicating loss of function [12]. Furthermore, the p.P600A dramatically affected the differentiation of patient-derived induced pluripotent stem cells into post-mitotic neurons, suggesting that fewer mature neurons may evolve during neuronal cell development in ATP1A1-CMT [33]. Taken together, alterations of the p.P600 residing at the bridge of the N and P domains of ATP1A1 damage the Na^+^/K^+^ ATPase activity and neuronal function.

Previously reported axonal CMT-*ATP1A1* variants; p.P600A and p.D811A demonstrated significant reduction in Na^+^ currents. The p.D811 is a predicted site of Na^+^ ion coordination suggesting that, missense changes would result in loss of sodium coordination [12]. Since p.P600A resides in the proposed molecular hinge domain, it was suggested that the reduced Na^+^-pump currents were due to impaired conformational dynamics of the pump during the Na^+^/K^+^ transport [12]. Conversely, our electrophysiology analysis on the demyelinating p.P600R variant demonstrated that the reduced pump function was due to reduced expression of the pump proteins instead of a kinetic dysfunction of the Na^+^/K^+^ ATPase. In addition, four axonal *ATP1A1* variants; p.L48R, p.I592T, p.P600T and p.D811A seem to have more detrimental loss of cell viability (more than two-fold decrease in viability compared to WT-ouabain sensitive cells) in the ouabain survival assay compared to our demyelinating p.P600A variant [12]. This may also reflect the differences in the cellular mechanisms of axonal variants versus the demyelinating p.P600R variant. Furthermore, the two previously identified CMTI mutants; p.S207F and p.G877S were shown to downregulate ATP1A1 protein expression by promoting proteasomal degradation of the mutants without affecting the mRNA levels [13]. Although the main mechanism of pathogenesis seems to be loss of function in all the reported types of *ATP1A1*-related CMT, the underlying molecular mechanisms for each type clearly have differences, warranting future studies to explore these mechanistic aspects in more detail.

Reduced Na^+^/K^+^ ATPase activity at the plasma membrane surface would eventually result in intracellular Na^+^ build up. The Na^+^/Ca^2+^ exchanger responds by reverting to move out excess Na^+^ while taking Ca^2+^ into the cytoplasm. This mechanism was previously implicated in various diseases associated with Na^+^/K^+^ ATPase mutations [34]. Mitochondria are essential for buffering intracellular Ca^2+^. Toxic elevation of Ca^2+^ will eventually lead to mitochondrial dysfunction resulting in increased production of reactive oxygen species (ROS). Mitochondrial ROS stimulates phosphorylation of the α1 subunit by protein kinase PKCζ, which triggers endocytosis of the plasma membrane Na^+^/K^+^ ATPase [35–38]. Phosphorylation-ubiquitination-recognition-endocytosis-degradation (PURED) is an established pathway for regulating plasma membrane proteins including the Na^+^/K^+^ ATPase [39–42]. Phosphorylation of the α1 subunit stimulates ubiquitylation under steady state and hypoxic conditions in alveolar cells and, it inhibits Na^+^/K^+^ ATPase in neuronal cells [43–45].

Hoxhaj et al., identified that the Thr339-Leu772 fragment of ATP1A1 is a binding site for the closely related E3 ubiquitin ligases; ZNRF1 and ZNRF2 [46]. ZNRF1 and ZNRF2 are expressed in many tissues including in the peripheral nervous system and are targeted to plasma membrane by *N*-myristoylation [46, 47]. The p.P600R that falls into the binding region of ZNRF1/2 on ATP1A1 might affect the binding of these ubiquitin ligases hence, degradation of the α1 subunit. Altering the degradation dynamics of the tightly controlled Na^+^/K^+^ ATPase activity might send a negative feedback message to downregulate the production of *α* and *β* transcripts. The ubiquitin–proteasome degradation pathway has been previously linked to the transcriptional regulation of proteins [48–50]. Nevertheless, such interplay between degradation and transcriptional regulation of Na^+^/K^+^ ATPase subunits needs to be clarified in future studies.

The variability in phenotype due to different amino acid substitutions of the same residue are likely to be due to the nature of the variant. Each substitution may have a different effect on the tertiary structure of the respective protein. Some amino acids would be more damaging due to variability in chemical structures and the bonds they may disrupt or introduce, resulting in altered protein function which may be reflected in the phenotype. For example, the Tyr82Cys variant in MPZ was described in patients with both CMT1 and Dejerine-Sottas Syndrome (DSS) and the Tyr82His change was identified in a milder late-onset phenotype CMT2 patient [51–54]. The Tyr to His change at position 82 of MPZ protein does not cause a significant structural change [54]. In contrast, introduction of the Cys residue at the same position has a large impact on the tertiary structure of the protein thus, on the myelin structure and function resulting in a more severe phenotype [54]. The ATP1A1 Pro600Arg change hereby presented in a CMT1 patient, likewise may have a different effect on the protein as compared to the Pro600Ala and Pro600Thr variants previously identified in CMT2 patients. Arginine has a larger structure with an electrically charged side-chain whereas, both Threonine and Alanine are smaller and lack an electrically charged side-chain. The wild type residue Proline is smaller than Arginine and has a special structure providing unique properties for the transmembrane proteins, unlikely to be recapitulated by any other amino acid with a different size.

## Conclusion

In conclusion, we identified a novel variant at the hotspot CMT residue P600 of *ATP1A1* using WES. The proband presented with demyelinating CMT in contrast to the previously identified P600A and P600T variants that cause the axonal type of the disease. Phenotypically, clinical presentation is similar to the previous cases reported [12], although the severity of the neuropathy, in this case, was more pronounced compared to other patients of comparable age group with *ATP1A1* variants (CMTNSv2 12–15 in 40–50-year-olds). Furthermore, previously reported upper limb MNCVs in affected patients ranged from 30 to 52 m/s, while our patient presented even lower velocities that were clearly in the demyelinating range. In addition, sensorineural hearing loss found in this patient has not been previously reported in *ATP1A1* associated phenotypes. Thus, the phenotypic spectrum of *ATP1A1*-related CMT appears to expand from the demyelinating to the axonal end of the spectrum, and this should be taken into consideration when investigating demyelinating CMT neuropathies.

## Supplementary Information

Below is the link to the electronic supplementary material.

Supplementary file1 (DOCX 15 KB)

## Data Availability

Data are available upon reasonable request.
