# Supplementary material for: The phenotypic spectrum of pathogenic ATP1A1 variants expands: the novel p.P600R substitution causes demyelinating Charcot–Marie–Tooth disease
Source: J Neurol. 2023 Feb 4;270(5):2576–90. doi: 10.1007/s00415-023-11581-w (PMC10130110; doi:10.1007/s00415-023-11581-w)
Supplement: Supplementary file 1 — Supplementary file1 (DOCX 15 KB) [file 415_2023_11581_MOESM1_ESM.docx]

**The phenotypic spectrum of pathogenic *ATP1A1* variants expands: the novel p.P600R substitution causes demyelinating Charcot-Marie-Tooth disease**

Feride Cinarli Yuksel^1^, Paschalis Nicolaou^1^, Kerri Spontarelli^2^, Maike F. Dohrn^3,4^, Adriana P. Rebelo^3^, Pantelitsa Koutsou^1^, Anthi Georghiou^1^, Pablo Artigas^2^, Stephan L. Züchner^3^, Kleopas A. Kleopa^5^, Kyproula Christodoulou^1^

^1^ Neurogenetics Department, The Cyprus Institute of Neurology and Genetics, 1683, Nicosia, Cyprus

^2^ Department of Cell Physiology and Molecular Biophysics, Center for Membrane Protein Research, Texas Tech University Health Sciences Center, Lubbock TX

^3^ Dr. John T. Macdonald Foundation, Department of Human Genetics and John P. Hussman Institute for Human Genomics, University of Miami, Miller School of Medicine, Miami, FL, USA

^4^ Department of Neurology, RWTH Aachen University Hospital, Aachen, Germany

^5^ Neuroscience Department and the Centre for Neuromuscular Disorders, The Cyprus Institute of Neurology and Genetics, 1683, Nicosia, Cyprus

Corresponding author: Kyproula Christodoulou (e-mail: [roula@cing.ac.cy](mailto:roula@cing.ac.cy))

**SUPPLEMENTARY INFORMATION**

**Table 1.** Primer sequences used in the qPCR cDNA expression analysis

| Primer | Sequence |
| --- | --- |
| ATP1A1_Exon_6_7F | ATGGCTGCAAGGTGGATAACT |
| ATP1A1_Exon_6_7R | GGCAATGTTCCTCGTCTCC |
| ATP1A1_Exon_14F | GGAGACCATCCAATCACAGC |
| ATP1A1_Exon_14R | CAATGTCTTCCACGGTCTCA |
| ATP1A1_Exon_16_17F | GTAGAGGAAGGTCGTCTGATC |
| ATP1A1_Exon_16_17R | CAGTGGTAGTGGAATGTTTGC |
| ATP1A1_Exon_21_23F | CTGGAATGGGTGTTGCTCTT |
| ATP1A1_Exon_21_23R | TTTCCTTCTCCACCCAGCC |
| ATP1B1_Exon_1_2F | CGGTGGCAGTTGGTTTAAGA |
| ATP1B1_Exon_1_2R | TCGGTCCTGATATGTGGGCT |
| ATP1B1_Exon_3_4F | AGATTGTGGCGATGTGCCC |
| ATP1B1_Exon_3_4R | GGTTTGCCCTCTTTGTAGCC |
| ATP1B1_Exon_5_6F | AAGCCTCCCAAGAATGAGTCC |
| ATP1B1_Exon_5_6R | CCTTATCTTCATCTCGCTTGCC |
| ACTINB_F | TCTACAATGAGCTGGGTGTG |
| ACTINB_R | AGAGGCGTACAGGGATAGCA |
| GAPDH_F | GGTCACCAGGGCTGCTTTTA |
| GAPDH_R | ACAAGCTTCCCGTTCTCAGC |
